# English Long and Short Sentence Translation and Recognition Method Based on Deep GLR Model

**DOI:** 10.1155/2022/3119477

**Published:** 2022-06-08

**Authors:** Hongmei Wang, Changhan Zhao

**Affiliations:** ^1^School of Foreign Studies, Wenzhou University, Zhejiang, Wenzhou 325035, China; ^2^School of Foreign Languages, Chongqing Technology and Business University, Chongqing 400067, China

## Abstract

The translation recognition of English long and short sentence information is an important issue to obtain the focus and core of English articles. Based on the deep GLR model, this paper constructs a method framework for English long and short sentence translation and recognition, using different embedding layer parameter initialization methods and using multi-layer computing methods in the sentence decoder. The initial corpus text is segmented and tagged with part-of-speech, then, the part-of-speech tag is appropriately corrected to reduce ambiguity, and then it is manually syntactically tagged. In the simulation process, the English long and short sentence summary and translation components are designed and developed, which can accurately and efficiently obtain the key information of English long and short sentences. The experimental results show that the English long and short sentence translation and recognition method of the deep GLR model improves the accuracy of the model parameters. In terms of model structure, the deep GLR value can be improved by 70.77% by reproducing the multi-layer representation fusion of semantic translation; in terms of data enhancement, the deep GLR value can be increased by 70.35% by means of “back translation,” and the improved model is effective. It promotes the translation and recognition generalization ability of English long and short sentences.

## 1. Introduction

In recent years, the development of modern information technology has enabled people to access and obtain a large number of information resources and English long and short sentences. The processing and translation of these English long and short sentences have further promoted the development of corpus linguistics [1]. At the same time, the development of corpus linguistics and the development of modern corpora have opened up new research directions for linguistics and other disciplines, and forensic linguistics, as a branch of linguistics, has good research prospects. Therefore, it is very important to summarize the information of long and short sentences in English and accurately translate it into Chinese to help people acquire the key points and core knowledge of English articles effectively and quickly. The task of automatic summarization of English long and short sentences is a challenging task in the field of natural language processing, and it is also a concern of many research teams. Most of the current models can calculate the correlation of abstract extraction or generation targets [2–4]. For supervised learning, the training data of abstracts are extracted to mark English long sentences and short sentences as abstract sentences, and the task is transformed into two categories: long sentences and short sentences; in the generative summary model, it is also a semantic unit with the marked artificial summary. For unsupervised learning, the modeling goal of most methods is the correlation between English long and short sentences and the content of English long and short sentences [5–7].

With the development of the Internet, widely available data are exploding, and human beings have entered the era of big data. A large amount of English information has brought information redundancy to users, and it has caused certain difficulties for users to browse and filter information. Based on the above research background, this study, as an empirical study, attempts to improve the practical problems of English translation of local regulations through a computer-aided translation model based on parallel corpus [8]. This research mainly focuses on the establishment of a corpus-based machine-assisted model, the translation quality assessment based on the model, and the scalability of the model in the local area network, the Internet, and the cloud. In the actual production environment, the use of similarity-targeted modeling methods with some artificial rules can usually meet the requirements. However, when translating longer English sentences, summarizing only for relevance will encounter a significant problem: the model will tend to generate a bunch of summary sentences with high similarity and lose some niche topics information. Although this information is not a high proportion of English long and short sentences, it is still important. Especially in English long and short sentences such as research reports or financial reports in the financial field, some important information does not appear many times in English long and short sentences [9–11].

First of all, corpus-based computer-assisted translation combines the advantages of speed and quality compared with human translation and machine translation, especially translation memory. Through the memory replacement function, assisted translators can achieve fast and efficient translation of English long and short sentences. The current situation of English translation of local laws and regulations has a positive effect. At the same time, the “Neural Machine Translation, Statistical Machine Translation, Vocabulary” translation system constructed in this paper can further improve the effectiveness of translation. Among them, neural machine translation uses transformer in tensor2tensor, and statistical machine translation uses IBM model in Moses. In order to quickly obtain the core content of English long and short sentences, this paper combines unsupervised machine learning TextRank to extract English long and short sentences, which can effectively extract the key information of English long and short sentences and translate English into Chinese more accurately through the translation system to help learners improve their learning efficiency. Secondly, although the corpus-based machine-assisted translation model is currently used to effectively improve the translation speed without affecting the translation quality as much as possible, the translation quality can be further improved by extending to the local area network, the Internet, and the cloud for application storage. After the open test, the accuracy and regression rate reached 80.8% and 74.3%, respectively, which were improved compared with the analysis results of the Prop analyzer.

## 2. Related Work

The goal of English long and short sentence summarization is to extract the most important information content from the original information and present it to the user, so it is very sensitive to the actual needs of the user. Users with different needs can obtain a different information from the same abstract, and even the same user will obtain a different information from the same abstract in different periods. Therefore, it is very difficult to evaluate the volume abstract. At present, most evaluation systems are highly targeted, and most of their evaluation methods are aimed at their own abstraction systems [12–14]. Wang et al. [15] believed that it is an extension of context-free grammar (CFG), and every rule and probability combination of CFG constitutes PCFG. Scholars from various countries have conducted in-depth research on PCFG, mainly in PCFG derivation and parameter learning. Li et al. [16] used the inside-outside algorithm to automatically estimate PCFG parameters for the first time and summarized various algorithms for translating PCFG with the bottom-up CYK algorithm, including the inside-outside algorithm and Viterbi algorithm; Wu et al. [17] combined the Earley algorithm with PCF, which gives a probabilistic version of Earley's algorithm. Although PCFG has the characteristics of concise form, small parameter space, and high analysis efficiency, and forms a relatively complete system, it ignores the context-related information necessary for disambiguation in the analysis, and the disambiguation ability is very limited. In response to the problems of PCFG, Ruano et al. [18] constructed a probabilistic model to increase structural information, a probabilistic grammar including lexical dependencies, and a history-based model. They are more effective for the expression of natural language, and these theories have played a certain role in promoting the research of syntactic analysis. Before the corpus syntax analysis method appeared, the research of syntax analysis was basically a rule-based method, and the acquisition of rules was a very cumbersome process, which completely depended on the language knowledge and experience of the knowledge engineer who developed the rules, even if it is also difficult for a trained language engineer to write grammatical rules that cover many linguistic phenomena.

Introducing complex features in probabilistic context-free grammars, Tolan et al. [19] stipulated that all rule preterms have only two nodes, that is, the rules in the binary form. Each nonterminal node is represented by a set of “attribute-value” pairs, allowing the representation of lexical unification relationships, as well as long-range dependencies. A dynamic domestication method is used in the syntactic analysis, and an unguided method can be used for parameter selection. Experiments show that this syntactic analysis method is effective. Mariyam et al. [20] introduced lexical dependencies into grammars and proposed a syntactic analysis method based on lexicalized probabilistic context-free grammars. Each nonterminal node in the rule is associated with its core word, and the dependency between the core words is reflected by the probability of the rule. The algorithm adopts the top-down PCFG syntax analysis method and makes some local improvements to PCFG. The syntactic analysis results obtained by Collins are the best results seen so far [21–24]. A history-based model is also described, which differs from Black in that Black's model uses a handwritten syntactic rule, while Jelinek's uses syntactic rules that are automatically trained from a corpus. Jelinek uses a bottom-up syntactic analysis process, and the selection of rules considers the sequence of parts of speech that constitute the rule, the nonterminal symbols generated by the rule, and the relationship between the child node and the parent node in the rule. The selection of rules uses a decision tree approach.

## 3. Pattern Recognition Based on Deep GLR Model

### 3.1. Deep GLR Model Hierarchy

The deep GLR model matches an English long and short sentence fragment in the reference sentence; then, this fragment cannot be matched again. So in the example, am has already been matched, so it can no longer be matched, so the new score = 1/5. At the same time, if there are multiple reference translations, an English long and short sentence fragment can only take the maximum number of occurrences in one reference sentence. For example, a appears 1 and 2 times in references 1 and 2, respectively, so take 2 instead of both.

(1) $$\begin{array}{c} \sum \text{count}\left(\text{click}\left(x,y\right),\text{click}\left(x-t,y-t\right)\right)\\ =\underset{x,i⟶\infty }{\lim}\left(\frac{1}{\text{click}\left(x,y\right)}-\frac{1}{\text{click}\left(x-t,y-t\right)}\right). \end{array}$$

**Figure 1:**
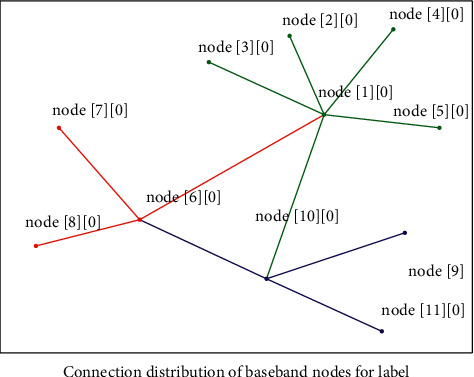
Node hierarchy of deep GLR model.

Assuming that there are *N* different points distributed on the two-dimensional plane of the depth GLR model, given a point among them, a Cartesian coordinate system is established. Based on this coordinate system, each point on it uniquely corresponds to a coordinate (*x*, *y*); then, introduce the Euclidean distance; finally, calculate the distance between the other *N* − 1 words of this word, and the word corresponding to the minimum distance value is the word we are looking for. According to the proposed method, “similar” words have similar eigenvectors, and since these eigenvalues are a smooth function for the probability function, a small change in the feature will only cause a small change in the probability. Therefore, the occurrence of any of the abovementioned English long and short sentences in the training data will increase the probability value, not only those English long and short sentences, as long as they are near the English long and short sentence space in Figure 1 (represented by the feature vector sequence). If a new model needs to be launched every time, it is only necessary to package the model and replace the original model, and the update iterative operation can be completed in a short period of time without affecting the use of various functions of the system for a long time.

By training a deep GLR model, each word in a language is mapped into a short vector of fixed length. Of course, the short here is relative to the long one-hot representation. Put all these ideas together to form a word vector space, and each vector is a point in this space. Introducing “distance” in this space, you can judge the similarity between words in terms of syntax, semantics, etc., according to the distance between them. In many cases, a pretranslation of the corpus is performed to generate an English long and short sentence of the corpus. In order to prevent the English long and short sentences from being too large, usually only words with a frequency greater than a certain threshold are thrown into the English long and short sentences, and all the remaining words are uniformly encoded as dunk. This is a very classic and simple approach, and this method cannot solve the problem of unregistered words.

(2) $$\begin{array}{c} \text{black}\left(bp.\;\exp\left(w\left(n\right)-bp.\;\log\left(n\right)\right)\right)-\frac{1}{bp.\;\exp\left(w\left(n\right)-bp.\;\log\left(n\right)\right)}=0. \end{array}$$

BPE English sentence pair encoding or binary encoding is a simple form of data compression in which the most common pair of consecutive bytes of data is replaced by English long sentences that do not exist in the data. A replacement table is required to reconstruct the original data for later use. The algorithm is described as a layer-by-layer iterative process in which the most frequent pair of characters in a string is replaced by a character that does not appear in that character. It has the advantage of effectively balancing vocabulary size and step count (the number of tokens required to encode long and short sentences in English).

### 3.2. Analysis of Deep Baseband Characteristics

**Figure 2:**
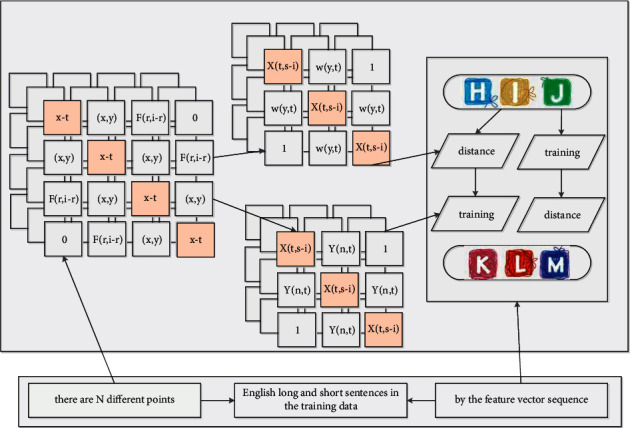
Deep GLR model framework topology.

Since the deep GLR algorithm needs complete information about the parameters of the identification signal, and in practice it is impossible for us to know all the information of the identification signal, the quasi-ideal deep GLR algorithm becomes more attractive. In general, the method of estimating the identification signal parameters can be adopted, and the estimated identification signal parameters are brought into the deep GLR algorithm. The network extraction system uses this seed set as the initial url to start data crawling. Because the webpage contains link information, some new urls will be obtained through the existing webpage urls. The pointing structure between webpages can be tried as a forest. The webpage corresponding to each seed url is the root of a tree in the forest. In this way, the web network extraction system can traverse all the webpages according to the breadth-first algorithm or the depth-first algorithm of Figure 2. Because the depth-first algorithm may make the extraction system fall into the interior of a website, which is not conducive to searching for webpage information that is relatively close to the homepage of the website, the breadth-first search algorithm is generally used to translate webpages.

The design idea of deep GLR is consistent with the idea of judging the quality of machine translation: the closer the machine translation result is to the result of professional human translation, the better. What the deep GLR algorithm is actually doing: two English long and short sentences, *s*1 and *s*2; the more words in *s*1 appear in *s*2, the more consistent the two English long and short sentences are. To judge the degree of similarity between two English long and short sentences, that is, to judge whether the meaning of an English long and short sentence is the same before and after the translation, obviously there is no direct comparison. The perplexity value of the test set is based on the probability value of each English long and short sentence in the test set. Using the formula, the probability of an English long and short sentence in the test set based on the n-gram grammar model can be obtained. For a test set *T* consisting of English long and short sentences, the probability *P*(*T*) of the test set can be calculated by calculating the product of the probabilities of all English long and short sentences in *T* and calculating the cross-entropy *H*(*p*) on the data *T* where WT is the number of English long and short sentences in the test set.

(3) $$\begin{array}{c} \left\{\frac{\text{lower}\left(m\left(x\left(t\right)\right),m\left(x\left(t-t^{\prime }\right)\right)\right)}{\text{higher}\left(m\left(x\left(t\right)\right),m\left(x\left(t-t^{\prime }\right)\right)\right)}\right\}⟶\left\{\frac{\text{low}\left(x\right)/x\left(t\right)}{\text{high}\left(x\right)/x\left(t\right)}\right\}. \end{array}$$

First of all, the activation function of the hidden layer includes four types identity, tanh, hard tanh, and rectifier in this experiment. We use these four activation functions to train the language model and find that the first three generated models contain a lot of NAN. The representative means if we add such a language model to the system for training, it means that the rectifier activation function is better than the other ones and meets our requirements. Therefore, the activation function used by default in all the following experiments is rectifier. When there are multiple convolutional layers in the neural network, the features extracted by the weight matrix are more and more complex and suitable for the target task. In the pooling layer, the maximum pooling is usually used, that is, taking the maximum value from a certain part of the input matrix and outputting it. Pooling layers can reduce the number of trainable parameters. The backend of the project is mainly developed based on flask, using requests to obtain user input and then performing a series of processing: including sentence segmentation, BPE word segmentation, and judging the type of input. Therefore, the matrix needs to be spread out, converted into a vector, and then passed through multiple fully connected layers (the same as the multi-layer before similar to the feed network) and output the probability of each class.

### 3.3. Semantic Stability Recognition

The semantic stability of English long and short sentences means that the computer analyzes the information of English long and short sentences, and selects English long and short sentences that can reflect the content of the English long and short sentences, or is generated by the computer on the basis of understanding the English long and short sentences in no redundant English long and short sentences. Users can obtain key information of English long and short sentences from the abstract without reading the entire English long and short sentences, thus helping users to improve reading efficiency. First, after setting the parameters related to deep GLR timing on the user interface, configure these parameters into each module in the FPGA through the deep GLR parameter configuration module. When the timing function is triggered, the baseband data and time stamp are generated by the frame splicing module. The write operation module reads the snapshot data from the FIFO and writes it to the DDR2 according to the operation timing of the DDR2 IP core. In the memory, the read process first passes through the mutual exclusion module to solve the problem of read and write priority, then the read operation module reads the data buffered in DDR2 into the transmit FIFO, and then the translation interface module transmits the snapshot data according to the translation timing to the DSP and finally is sent to the computer by the Ethernet port.

(4) $$\begin{array}{c} \frac{\text{count}\left(\text{click}\left(x,y\right)\right)}{\text{count}\left(\text{click}\left(x-t,y-t\right)\right)}=\frac{\sum \text{count}\left(\text{click}\left(x,y\right)\right)}{\sum \text{count}\left(\text{click}\left(x-t,y-t\right)\right)}. \end{array}$$

It can be found that a length penalty factor is added to the semantic stability. Because of a long and short sentence in English, as long as a small part of the translation is guaranteed to be correct, the score will still be high. Therefore, the matching degree of n-gram may become better as the length of English long and short sentences becomes shorter, which will lead to such a problem: a translation engine only translates some English long and short sentences in English long and short sentences, the translation is more accurate, and then its matching will still be high. To avoid this scoring bias, deep GLR introduces a length penalty factor into the final scoring result. This model of split-translating a sequence of words, considering only a finite history of states at each step, is called a Markov chain. Usually, the number of historical vocabulary needs to be selected considering the size of the training corpus. When the size of the training corpus is large enough, a longer history can be selected. In general, the ternary grammar language model is used. The model uses the history of the two words to obtain the third word, which requires counting all sequences of three words. Taking the binary grammar as an example, the predicted probability *w*1 of the word is estimated according to the maximum likelihood method as shown in the text. Just count the number of times that word *w*2 follows *w*1 in the training corpus and the number of times that other words follow *w*1.

(5) $$\begin{array}{c} \text{sum}\left(\Delta t\left(pt\right)|t\left(x,y\right)=n\right)=\frac{\sum \text{count}\left(pt-n\right)}{\text{count}\left(\ln\left(x,y\right)\right)}. \end{array}$$

The encoder encodes the input sequence into a fixed-length vector *C* through nonlinear transformation, which represents the semantic information of the input sequence, and then the decoder decodes and analyzes the vector to output a sequence of variable length (the decoder is usually a neural network). This operation process is actually similar to the error correction process of English long and short sentences; that is, it is necessary to first understand the semantic information to be expressed by the wrong English long and short sentences, and then reorganize the language to form correct sentences according to the semantic information and the structure of English long and short sentences. The context vector *C* is the final state output by the encoder, usually the last hidden state in the depth GLR or the weighted sum of multiple hidden states, which is used as the initial state of the decoder. 0*y* is the final output character of the encoder, which is generally a termination symbol, which means that the input sequence in Table 1 has been read, and is used as the initial input of the decoder, which represents the start of decoding to generate an output sequence until it encounters the termination symbol.

Then, decide whether to obtain the translation result from the word list, phrase vocabulary, statistical machine translation, or neural machine translation, and then take certain processing and return the translation result to the user. The set of relations for each language is the “language space,” and this “language space” is represented as a vector set space. In the vector set space, there are many commonalities between different languages. As long as the projection and mapping of one vector space to another vector space are realized, the translation between the two languages can be realized. In order to better understand this process, a special example is as follows: consider two languages, English and Chinese, and obtain their corresponding word vectors *E* and *C* through training. Take five words one, two, three, four, and five from English, and set their corresponding word vectors in *E* to be *v*1, respectively. For the convenience of drawing, use principal component analysis (PCA) to reduce the dimension, get corresponding two-dimensional vectors *u*1, and trace these five points on the two-dimensional plane. Similarly, take out one, two, three, four, and five in Chinese, set their corresponding word vectors in *C* to be *s*1, the two-dimensional vectors after PCA dimension reduction are *t*1, and trace them on a two-dimensional plane, possibly with proper rotation.

### 3.4. Model Weight Recursion

First, the query and key in the weight of the model are calculated by dot product, then normalized by softmax, and then multiplied by the weight, respectively, and the attention vector is obtained by accumulating. The operation between query and key is equivalent to calculating the internal similarity of the input sequence, and according to this similarity or weight, the internal relationship of the sequence itself (value) is noticed. This internal connection may be that the subject notices the information of the predicate and the object or other structures hidden inside the English long and short sentences. This can be linked to the previous methods of implementing attention; that is, they all use similar methods, but use different implementation forms, which can be additive or multiplicative.

(6) $$\begin{array}{c} \text{Attention}\left(k,j\right)-\text{steel}\left(k,j\right)=\langle \begin{array}{c} \frac{\Delta t\left(k,j\right)}{\ln\left(k,j\right)},k>j\\ \frac{\Delta t\left(pt-k,pt-j\right)}{\ln\left(1,j\right)},k<j \end{array}. \end{array}$$

The bit width of the translation address instruction is 32 bits, the format is 32′hABCDXXXX, the upper 16 bits are 16′hABCD which is the translation address instruction flag bit, and the lower 16 bits are the instruction translation address number, which is used to distinguish which type of control identification signal it is. For example, 32′hABCD0601 represents the instruction to configure the timing trigger time of the deep GLR. The content of the content instruction is the specific required configuration parameters, such as the specific trigger time. Seq2seq is a commonly used model for translating sequence data. Since the input and output of the seq2seq model are sequences, the model can be widely used in natural language translation tasks, such as machine translation and automatic summarization of English long and short sentences.

**Figure 3:**
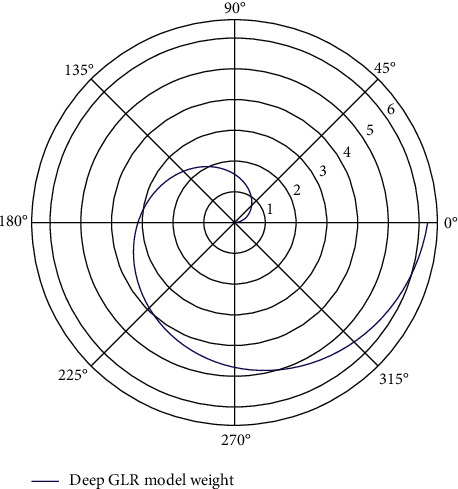
Weight polarization distribution of deep GLR model.

Model weight for each nonleaf node is shown in Figure 3; it is necessary to specify a class for its left and right child nodes, that is, which is the positive class (labeled as 1) and which is the negative class (labeled as 0). As it happens, every node in the tree, except the root node, corresponds to a Huffman code with a value of 0 or 1. Therefore, a natural approach is to define the node with Huffman coding as 1 as the positive class, and the node with the Huffman coding as 0 as the negative class. However, this method of definition is only a convention, and it can also be coded as A node with a 1 is defined as the negative class, and a node with a code of 0 is defined as the positive class. When the user scrolls in English, Chinese should scroll with it and vice versa. Or, the format of the translated Chinese should be aligned with the format of the input English, such as line breaks.

(7) $$\begin{array}{c} \frac{\Delta t\left(pt\right)+\Delta t\left(pt-1\right)}{\Delta t\left(pt\right)-\Delta t\left(pt-1\right)}-\max\left(t,t-1\right)=0. \end{array}$$

Therefore, it can be seen from this example that for any word in the dictionary *D*, there must be a path *p* − *w* from the root node to the corresponding node of the word *w* in the Huffman tree, and this path is unique. There are *w* branches on the path *p* *w*, and each branch is regarded as two classifications, each classification will generate a probability, and multiplying these probabilities is the required *p* (*w*|Context (*w*)). Finally, it is fed back to the PC host computer. There are mainly three status identification signals in this system, namely, GPS_READY, DDR2_READY, and GPS_TMIE_VALID. If the deep GLR receiver is in normal working state, that is, the number of received particles meets the requirements, and the pulse-per-second identification signal is accurate and stable, then GPS_READY is high; DDR2_READY means that the snapshot storage function is normal; GPS_TMIE_VALID means that the set trigger time format is consistent with required.

## 4. Construction of English Long and Short Sentence Translation and Recognition Model Based on Deep GLR Model

### 4.1. Synchronization of Deep GLR Model Information Flow

The deep GLR model information flow extraction system first puts the seed url into the download queue and then simply takes a url from the head of the queue to download its corresponding webpage. After the content of the webpage is obtained and stored, some new urls can be obtained by parsing the link information in the webpage, and these urls can be added to the download queue. Then, take out a url, download its corresponding webpage, then parse it, and so on; until the entire network is traversed or a certain condition is met, it will stop. For the Skip-gram model, the current word *w* is known, and the word Context (*w*) needs to be predicted. The form of the objective function corresponds to the objective function of the CBOW model, and the key is the conditional probability function *p*. The construction of (Context (*w*)|*w*) is defined as below in the Skip-gram model.

(8) $$\begin{array}{c} \text{softmax}\left\{\frac{\Delta t\left(pt\right)+\Delta t\left(pt-1\right)}{\sqrt{\Delta t\left(pt\right)-\Delta t\left(pt-1\right)}},\frac{\Delta q\left(kt\right)+\Delta q\left(kt-1\right)}{\sqrt{\Delta q\left(kt\right)-\Delta q\left(kt-1\right)}}\right\}\\ \subseteq R\left(\Delta q\left(kt\right)\cup \Delta q\left(kt-1\right)\right). \end{array}$$

The reason why only the content of the remaining words except the first word is stored is because the number of the word list can be used to calculate what the first word is, and all words in this word list start with this word. Often, however, the translation process utilizes only the top layers of the encoder and decoder, which misses the opportunity to utilize useful information in other layers. According to the model proposed by it, this system does multi-layer representation fusion based on the transformer model in tensor2tensor, which can basically reproduce the effect of the paper. When the second pulse identification signal pps_syn arrives, the counter value is reset to 0 and starts counting again, then the value of standard fre_next is updated to the total value of the previous second 180000004, and then the second pulse identification signal is synchronized to the baseband data of Table 2. The identification signal field generates the time ns_en_syn identification signal, and when the rising edge of the identification signal arrives, the current count value of the counter is recorded, thereby completing the translation and counting of nanosecond time.

When the trigger mode of the *G* timing service is timing trigger and level continuous trigger, since the time stamp second pulse is obtained through the synchronization of the *G* second pulse, they are relatively close in time, that is to say, the current calculated value ix of the counter is relatively small, it can also be seen from it, so the counting error caused by the offset of the crystal identification signal is also very small and can be ignored. The time stamp accuracy in these two trigger modes is relatively high, and the error can be guaranteed within 50 ns, while in the burst single trigger mode, the position of the time stamp is related to the burst mark identification signal, rather than the synchronization of the second pulse.

### 4.2. English Long and Short Sentence Translation Parameter Configuration

In the English long and short sentence translation parameter setting, when using softmax for normalization, the experiment uses two experimental methods: log-likelihood log and noise contrast estimation NCE. The experimental results show that the speed of NCE is much faster than the speed of log, and the multiple of the speed is 15 times more, which is useful for long-running experiments, verifying that NCE can avoid repeated summations in the log, greatly reducing the running time. S is used to constrain the size of the dot product. When the dimensions of query and key are large, the dimension of the result of the dot product will be relatively large, so the above factors need to be constrained. That is, the factor plays a regulating role so that the inner product is not too large. After the multi-head operation is completed, add an Add & Norm and then a feed-forward neural network. This is an encoder section in which six identical encoders are stacked as a whole. In the decoding part, self-attention will also be done first, and then the encoding and decoding part of the multi-head will be done. Here, *Q* ≠ *K* = *V*, and only self-attention is *Q* = *K* = *V*. Also in the decoding part, it is necessary to do a masked (the word after masking).

(9) $$\begin{array}{c} \text{Bpnetwork}\left(r,t\right)=\left\{\begin{array}{cc} xp\left(\frac{-\text{sig}\left(r+t\right)}{\text{sig}\left(r-t\right)}\right), & er<t\\ \frac{1-\exp\left(r+t\right)}{\exp\left(r-t\right)}, & r>t. \end{array}\right.. \end{array}$$

It can be seen that both uLSIF and depth GLR can approximate the density ratio very well and the influence of the excellent relative coefficient of depth GLR, and the density ratio is also smoother and can better approximate the smoother density ratio function, which is consistent with the previous discussion. In the experiment, the equivalent baseband BPSK system is used to identify the signal. The length of the signal segment is 100 sample points (number of symbols), the length of the noise segment is 100 sample points, 50 identification signal modules, then the sudden change point of the burst identification signal is unknown as *t* = 100 : 100 : 9900, the identification signal rate is 10 dB, and the number of experiments is 50 times. Through a matrix model, the translation unit and the original language, thinking, and cultural level are recorded, and the ambiguity of the original text is finally calculated. On this basis, considering the factors closely related to the translation of the fuzzy original text and the reference of the nonlinear formula, that is, “M1.v1 + M2 oV2 + M3 oV3 = U′S score” is a reasonable translation method for articles with fuzzy features.

**Figure 4:**
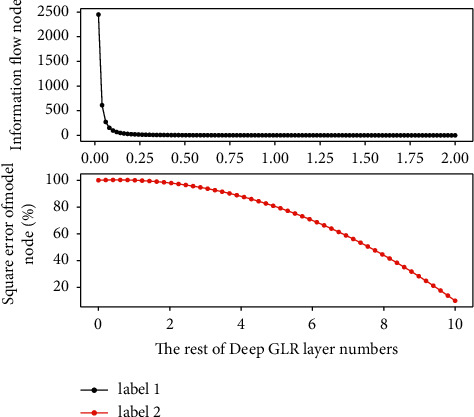
Feedback of deep GLR model information flow.

Here, a hardware windowing method is used to eliminate the influence of the pseudo-second pulse in Figure 4. By judging whether the count value of the counter is within the range of 180 t when it receives the second pulse identification signal, the anti-interference of the second pulse is realized. This method is equivalent to adding a high-level window to the range of 180 t. When the rising edge of the second pulse identification signal falls within this window, it is the correct second pulse identification signal, and the identification signal that does not fall within this window will be filtered out. By reasonably setting the size of the window parameter *t*, the anti-interference effect can be adjusted. This design set *t* is to 100.

(10) $$\begin{array}{c} H^{\prime \prime }\sqrt{\frac{x{\left(i\right)}^{2}-x{\left(j\right)}^{2}}{\max\left(x\left(i\right),x\left(j\right)\right)}}-H^{\prime }\sqrt{\frac{x{\left(i\right)}^{2}+x{\left(j\right)}^{2}}{\max\left(x\left(i\right),x\left(j\right)\right)}}=H^{\prime \prime }-H^{\prime }\left(\sqrt{x\left(i\right)+x\left(j\right)}\right). \end{array}$$

The deep GLR structure is very suitable for modeling sequences, which is more in line with the order that humans understand English long and short sentences (from left to right), and the required memory size is only related to the size of the vocabulary and does not grow exponentially with the length of the context. Combining the input *t x* at time *t* and the output *t* − 1 h of the hidden layer at the previous time, the hidden state *t* h at the current time *t* is calculated, and then the probability of the next word appearing is calculated according to *t* h. The perplexity is essentially standardizing the probability of the target English long and short sentences, that is, the average branch factor, which is used to predict how many choices there are for the next word. For example, if the perplexity is 5, the language model is generating a sequence, and there are 5 reasonable choices for the next word. If the number of optional words is smaller, it can be roughly considered that the language model is more accurate; that is, the smaller the perplexity, the better the language model.

### 4.3. Identifying Model State Feedback

After the user enters the text, the entire process from reading the user's data, to sending the data to the model, and then returning the result generated by the model to the user should not take too long. In the case of low identification signal rate, the deviation of the symbol interfered by noise from the real symbol is larger than that in the case of high identification signal rate; then in the case of low identification signal rate, we need to smooth more constellation points in order to determine the real symbol; in the worst case, we need to smooth all constellation points, and in the case of high identification signal rate, only a few closest points need to be smoothed. That is, in the best case, we even only need to smooth one or two points closest to the received symbol, which greatly reduces the amount of computation and improves the performance of the maximum likelihood recognition algorithm. Transformer's encoder consists of a stack of 6 identical layers, each with two sub-layers; the first is a multi-head self-attention mechanism, and the second is a position feed-forward neural network, in each of the two sub-layers. After that, there are residual connections and normalization operations; the decoder is also composed of 6 stacks of the same layer; except for the two sub-layers of the encoder, the encoder inserts the masked multi-head self-attention between the two sub-layers; multi-head attention is a new concept proposed by Google, which is the improvement of the attention mechanism. Formally, it is to map *Q*, *K*, and *V* through the parameter matrix, and then do attention, repeat this process *h* times, and splice the results together.

(11) $$\begin{array}{c} w\left(\text{max}\left(i\right),\text{max}\left(j\right)\right)=\sqrt{\frac{1}{N}\sum \frac{x{\left(i\right)}^{2}+x{\left(j\right)}^{2}}{\max\left(x\left(i\right),x\left(j\right)\right)}}+\sqrt{\frac{1}{N}\sum \frac{x{\left(i\right)}^{2}-x{\left(j\right)}^{2}}{\min\left(x\left(i\right),x\left(j\right)\right)}}. \end{array}$$

**Figure 5:**
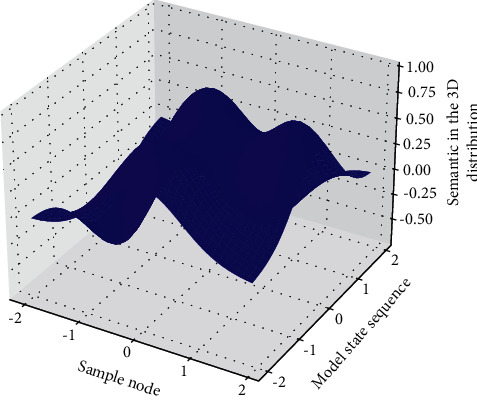
Three-dimensional distribution of the state sequence of the recognition model.

This subsection observes the performance of the deep GLR recognition signal rate estimator through experimental simulations. The experimental parameters are set as follows: oversampling rate Nss = 16, root raised cosine filter 128-order roll-off coefficient *A* = 0.5, number of symbols 1024, and the modulation type of the simulated identification signal is BPSK identification signal. It can be seen that in the case of low identification signal rate, the deviation of RxDA estimator is relatively large relative to CRB and TxDA, which is caused by the error of symbol decision in the case of low identification signal rate; when the identification signal rate increases, the reduction of the symbol decision error also reduces the mean square error of RxDA, which is almost the same as that of TxDA. Since the identification signal information is fully known by TxDA, the MSE curve of TxDA basically coincides with the CRB. It can also be seen from the deviation surface in Figure 5 that the large deviation in the case of low recognition signal rate also causes the increase of MSE.

However, in actual calculation, if the length of the language sequence is long, the parameter space of the language model is too large, and the estimation of the conditional probability *P*(*w*_*i*_*|w*_1_, *w*_2_,…, *w*_*i*−1_) will be very difficult and cannot be useful. In order to solve the problem that the parameter space is too large, the Markov assumption is introduced; that is, it is assumed that the probability of a word appearing is only related to the limited one or a few words that appear before it. From this, a simplified model is proposed: the *N*-gram model. When estimating conditional probabilities in an *n*-gram language model, only the first *n* − 1 words of the current word are considered. Among them, count(s) represents the number of times the sequence *s* appears in the training set. The words in the vocabulary are stored in the ascending order of the pinyin of the words, and the vocabulary is also stored in the ascending order of the pinyin of the first word. There are a total of 6768 such vocabularies (data blocks) in the lexical analysis system.

(12) $$\begin{array}{c} \frac{\text{count}\left(u\left(i,n\right),u\left(i,n-1\right),\ldots ,u\left(i,n-t\right)\right)}{\text{count}\left(v\left(i,n\right),v\left(i,n-1\right),\ldots ,v\left(i,n-t\right)\right)}=\lim\frac{\text{count}\left(u\left(i,n\right)-u\left(i,n-t\right)\right)}{\text{count}\left(v\left(i,n\right)-v\left(i,n-t\right)\right)}. \end{array}$$

First give the first *n* − 1 words a vector in the continuous space (find the vector of the corresponding index in the matrix *C*), then splice the *n* − 1 word vectors, then pass the tanh activation function, and finally use the softmax function to calculate the probability distribution of the next word, using a neural network to model the constrained relationship between the probability of the current word and its previous *n* − 1 words. This model learns the distributed representation of words through neural network, which is not only low-dimensional and compact, but also contains semantics, which lays the foundation for the commonly used word vectors. Obviously, this method has better generalization ability than *n*-gram.

## 5. Application and Analysis of English Long and Short Sentence Translation and Recognition Model Based on Deep GLR Model

### 5.1. Deep GLR Model Data Pretranslation

Global attention has an obvious disadvantage: when predicting each target English sentence, it must pay attention to all English sentences in the encoder, which is very expensive and impractical when the input sequence is relatively long. For this problem, we selectively focus on some words in the input sequence. This method effectively avoids the expensive computation in global attention and is easier to train. The constituent elements of source are composed of a series of <key, value> data pairs. Given an element query in target, the attention calculation process is divided into three stages: calculating the similarity or correlation between the query and each key. In the second stage, the softmax operation is performed to obtain the weight coefficient of each key corresponding to the value; in the third stage, the weighted summation of the value is performed; that is, the final attention value is obtained. So in essence, the attention mechanism is a weighted summation of the values of the elements in the source, and the query and key are used to calculate the weight coefficient of the corresponding value.

(13) $$\begin{array}{c} \sum_{i,s<t-i}\frac{T\left(i,ns\right)}{T\left(i,ns\right)-1}-\sum_{i,i-1}\frac{x\left(i\right)-1}{x\left(i-1\right)-1}=\sum \exp\left(-s-t\right). \end{array}$$

**Figure 6:**
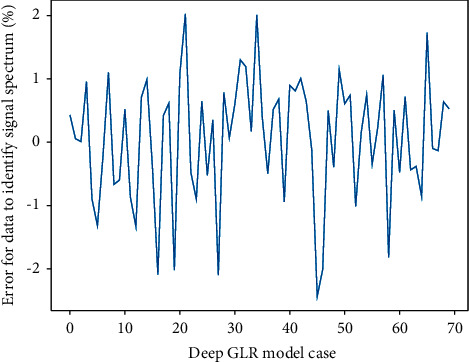
Deep GLR model data recognition signal spectrum.

For the first flip-flop Q1, connect VCC to its data input terminal, and connect the second pulse identification signal pps_syn of the count identification signal field to its identification signal input terminal, while the identification signals of the other two flip-flops are identification signal clk_ddc of baseband data. When the pulse-per-second identification signal pps_syn arrives, Q1 is driven high, and this value is transferred to the final time stamp pulse identification signal stamp_pluse after the next two identification signal cycles. The first step is to calculate all the rules to generate a set of items, each item is actually a state, and there is a certain connection between them, which is represented by a state transition diagram in this paper. Then, the attention weight vector is softmax normalized and linearly weighted with the information value at all times of the input sequence to obtain the attention vector. Obviously, in the same sequence of English long and short sentences, query = key = value, self-attention enables each word in the sequence of Figure 6 to establish a sensitive relationship with other words at any distance in the sequence, which to a certain extent raises the upper limit of the ability to model long-distance semantic dependencies.

The second step is to construct a GOTO and ACTION analysis table based on this state transition diagram. From the decision variables of the maximum likelihood algorithm, it can be seen that the reason for the large computational complexity of the algorithm is the smooth translation of the sample probability density function, and the smoothing of the probability density for a constellation of size Mk directly leads to Mk times the computational complexity of the algorithm. The increase is because the symbol N-S actually transmitted is determined by only one symbol in the constellation, but due to the interference of noise, at the receiving end we cannot determine which symbol is transmitted, and according to the minimum error rate criterion, assuming that all symbols in the constellation are transmitted with equal probability, we get our maximum likelihood identifier. Although the maximum likelihood discriminator is derived from the minimum error rate criterion, the assumptions here are still too conservative.

When the frequency of the identification signal is 60 MHz, the error is maintained within 100 ns; when the frequency of the identification signal is increased to 120 MHz and 180 MHz, the time error is significantly reduced and basically maintained within 50 ns, which is an acceptable range for time difference estimation. After the frequency is increased to 240 MHz, the fluctuation range of the error increases significantly, and the maximum has reached 687.5 ns. This is because the identification signal frequency is too high, which leads to metastability, which makes the data wrong in the sampling process. Through the research on the metastable state and the analysis of the measured data, it can be seen that the frequency of the identification signal that is too high and the frequency of the identification signal that is too low may lead to the increase of the timing error. In this system, the frequency of the counting identification signal is 180 MHz.

(14) $$\begin{array}{c} \left.\begin{array}{c} \frac{f\left(w\left(x\right)-w\left(x-t\right)\right)-f\left(w\left(x\right)\right)}{f\left(w\left(t\right)\right)}\\ f\left(\frac{w\left(x\right)-w\left(x-t\right)}{w\left(x\right)}-\frac{w\left(x\right)-w\left(x-t\right)}{w\left(x-t\right)}\right) \end{array}\right\}=h\left(w,x\left(t\right)\right). \end{array}$$

In translation and summarization tasks, only some words in the input sequence are relevant to the prediction of the next word; in picture description problems, some regions of the input image may have strong correlations with descriptors. After these two tables are constructed once, as long as the rule set does not change, they can be reused in the future syntactic analysis, without the need to construct the itemset and the analysis table. It is worth noting that the optimal solution *c* is not 0. From this, we can see that when the real MSE expression is optimized, the constant term that appears may be able to improve the performance of the modified estimator. It can be inferred from the closed solution expression that when *N* is large, the deep GLR will converge to the MLE. When we assume that the modified version is a linear expression of MLE, we get the same conclusion as deep GLR. It can be seen from the simulation experiments in the following that *c* is not reflected in MSE, indicating that *c* does not have much effect on improving performance in practice.

### 5.2. Statement Recognition Simulation Implementation

The basic English seq2seq model in the encoding phase simply converts the input sequence into a fixed-length vector, which is then decoded into the target sequence. It can be seen that there is no difference in the semantic encoding *C* of the input sequence *X* used in the process of generating all words in the decoding stage. This means that no matter which word is generated, any word in the input sequence *X* has the same influence on generating a target word, but in fact this is not equal weight.

(15) $$\begin{array}{c} \arg\max\left\{\prod_{x,y}^{p\left(x,y\right)}p\left(x\left(i\right),y\left(i\right)\right)|\left\{\frac{x\left(0\right),x\left(1\right),\ldots x\left(i\right)}{y\left(0\right),y\left(1\right),\ldots y\left(i\right)},\max\left\{x\left(i\right),y\left(i\right)\right\}\right\}\right\}=1. \end{array}$$

**Figure 7:**
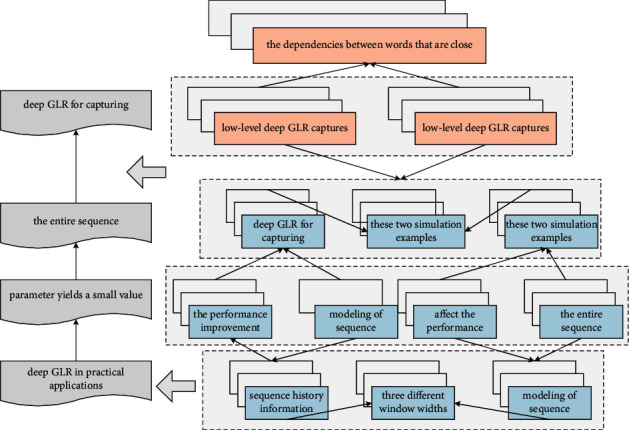
Recognizing nesting of long and short sentences in English.

The low-level deep GLR captures the dependencies between words that are close together, and the high-level deep GLR captures the dependencies between distant words. Through the hierarchical structure, a function similar to deep GLR for capturing the dependencies of sequences with a length of more than 20 words is achieved. The modeling of sequence by deep GLR relies on the historical information of the sequence, so it cannot be implemented in parallel. In contrast, the layered depth GLR convolves the entire sequence without relying on sequence history information and can be implemented in parallel, especially in industrial production; when faced with large amounts of translation data and high real-time requirements, the model training is faster and more efficient and high. It is clear that the modified estimator outperforms MLE for all SNRs, and the performance improvement is more pronounced in the small data case. It is observed that the MSE curve of the depth GLR coincides with the MSE curve of the LMMLE, which indicates that the constant term in Figure 7 does not play a significant role in practical applications in the unconstrained case. In the latter treebank, only sentences appearing V–V or N–N are included, and their internal detailed structures are annotated according to the rules of HDSM.

In these two simulation examples, each simulation takes a sample size of *n* = 200 and is repeated 100 times. The estimation of the unknown parameter *a*_0_ in the two cases is shown in the text. In the two cases of known and unknown *Q*, we give the profile least squares estimation of the stroke in example 1 and example 2, and in order to show the profile least squares estimation of the wind estimation is not sensitive to the choice of window width, we considered three different window widths.

The node_number represents the node number, node_name represents the node name, sons_number is an integer array with 12 elements, which is used to store the numbers of all child nodes of this node, and the pointer next points to the next node. It can be clearly seen from the various tables that all the optimization schemes in this chapter can effectively improve the depth GLR value: using “back translation” data, deep GLR increased by 0.5%; using adjusted alpha value, deep GLR increased by 0.7%; using adjusted beamsearch, deep GLR increased by 0.2%; using model fusion, deep GLR increased by 0.4%. According to the transformer multi-layer representation fusion reproduced in this paper, that is, by implementing iterative aggregation and hierarchical aggregation methods, respectively, the deep GLR is improved by 0.42% and 0.77%, respectively.

(16) $$\begin{array}{c} \text{weekset}\left(u\left(i\right),v\left(i\right)\right)=\langle \begin{array}{c} \frac{1-u\left(i\right)}{1-d}\\ -\cos\left(1+d\right) \end{array}|\begin{array}{c} \cos\left(1-d\right)\\ \frac{1+u\left(i\right)}{1-d} \end{array}\rangle . \end{array}$$

For each given 0 value in example 1 and example 2, we use the conditional bootstrap method to give 1000 Monte Carlo simulations to calculate the critical value of the GLR test statistic, and for three different test levels, Ga = 0.01, 0.05, and 0.10 are calculated from 500 simulations of 200 observations, respectively, and the rejection frequency is calculated. When 0 = 0, under different error distribution conditions, the efficacy of the next three different tests of water armor is (0.012, 0.014, 0.009), (0.044, 0.057, 0.053), and (0.053) in example 1; in example 2, (0.014, 0.012, 0.012), (0.046, 0.058, 0.046), and (0.104, 0.110, 0.080). This shows that the conditional bootstrap method gives the correct test level. This again shows that the GLR test not only has high power when the null hypothesis is far from the alternative hypothesis, but also shows that it has certain robustness under different error distributions to a certain extent.

**Figure 8:**
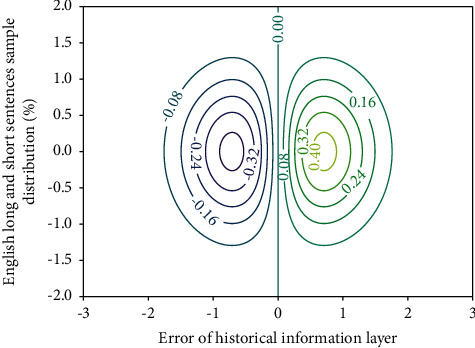
Historical information distribution of English long and short sentence sequences.

First find *P*(*r*) for each usage rule, and multiply this *P*(*r*) by *P*(*T*) in the process. Intuitively, whenever deep GLR is designed for *i*, we do not expect it to have better resolution than Figure 8 in the range of 6 dB. Therefore, the maximum optimization degree in the deep GLR case is larger than that in the 2S case, because the latter emphasizes one more possibility. However, merely explaining the worst-case probability does not determine the performance in the specified interval. This means that the deep GLR in the case of deep GLR does not necessarily have to be consistently better than 2S, which can also be seen from the figure, although deep GLR is still better than 2S in most areas. We can infer that there is an inherent trade-off between worst-case MSE and the best achievable performance, but this also cannot be used to specifically describe performance within regions.

### 5.3. Example Application and Analysis

The workflow of the entire English long and short sentence translation recognition system can be briefly described as follows: first, the user sets various parameters related to timing on the PC interface according to the specific test requirements and then uses the network interface to send these parameter instructions to the DSP system, and the DSP acts as the network interface. At the same time, it should be noted that the symbol stack processed in a typical GLR algorithm corresponds to the part-of-speech tagging symbol stack. If the timing function is enabled, the snapshot data will be sequentially cached in the DDR2 memory, and then the snapshot data will be sent back to the host computer through the DSP for the next step. In addition, parameters such as F bandwidth and center frequency in digital down-conversion (DDC) are also configured in this way.

(17) $$\begin{array}{c} \frac{\sum_{i,j}w\left(i\right)+w\left(j\right)}{\sum_{i,j<1}\mathrm{cov}\left\{w\left(i\right)+w\left(j\right)\right\}/w\left(i\right)+w\left(j\right)}\leq \\ \frac{\sum_{i,j}w\left(i\right)+w\left(j\right)}{\sum_{i,j<1}\mathrm{cov}\left\{w\left(i\right)\right\}/w\left(i\right)+\sum_{i,j<1}\mathrm{cov}\left\{w\left(i\right)\right\}/w\left(i\right)}. \end{array}$$

Although increasing the frequency of the identification signal can improve the time resolution of the counter and the accuracy of timing, blindly increasing the frequency of the identification signal will cause the flip-flop to encounter metastable problems during the synchronization process, resulting in data sampling errors. Therefore, a reasonable selection of the identification signal frequency of the timer is the key to improving the time stamp accuracy and the stability of the snapshot system. The metastable state usually occurs when the flip-flop is sampled. If the setup and hold time of the flip-flop cannot be met in an asynchronous design, the metastable state will be entered. In this state, the output level of the flip-flop cannot predict whether it is a high or low level, and may also hover between high and low for a period of time. Even worse, the metastable state will continue to follow the cascade of flip-flops.

**Figure 9:**
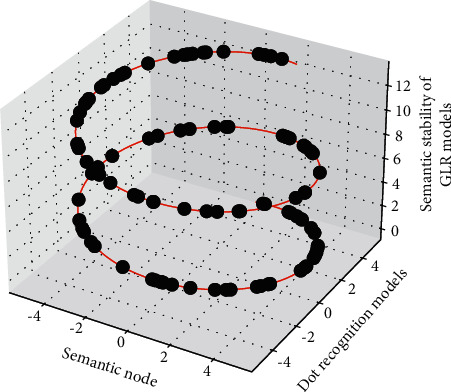
Semantic recognition system stability of deep GLR model.

For each given port, we use the bootstrap test to obtain 1000 Monte Carlo repetitions to calculate the test critical value in Figure 9 and then calculate the rejection frequency with 500 repetitions. Here, we consider two cases of sample size of 100 and 200, and the test level is considered separately for *c* = 0.01, 0.05, 0.10. When the sample size is *n* = 200, under different distributions and different test levels, the first type error frequencies are (0.010, 0.010, 0.015), (0.050, 0.046, 0.015), respectively, and (0.125, 0.116, 0.086), which indicates that the bootstrap test gives a better test level. When the sample size *n* = 100, the same superior test level was obtained from the paper, and all the power calculation results are shown in the text. It can be seen from the simulation results that the power of the test increases rapidly with the increase of the *p* value and shows a relatively robust growth level for the different error distributions in Table 3, which shows that the GLR test shows better results to a certain extent.

This article also needs to know the words corresponding to the part of speech, so this article adds a new stack, denoted as WordsStack, to store the words corresponding to the part-of-speech symbol in the part-of-speech tag stack. Assuming that *i* is mapped to 1.1, then *f* (1) = *s* before the residual is introduced, *h* (1) = *q* after the residual is introduced, and *h* (1, 1) = *r*. It can be clearly seen that the mapping after the introduction of residual connections is more sensitive to changes in the output. For example, when the output changes from 1.1 to 1.2, for a network without residual structure, the mapping *f* changes from 1.1 to 1.2, an increase of 1/11 = 9%, while for a network with residual structure, the mapping *f* is from 0.1 to 0.2, an increase of 1/11 = 9%. Therefore, the network with residual connections has a greater effect on the adjustment of parameters and can learn parameters with better effects. The model ensemble can make full use of the diversity between individual learners and comprehensively consider the error correction information from different angles, which improves the error correction performance by 0.86.

## 6. Conclusion

In the deep GLR-based model, the embedding layer parameters in this paper are usually initialized with pretrained English long and short sentence word vectors. At the same time, the probability of the rule needs to be obtained by analyzing the marked corpus. The incompleteness of the marked corpus also causes the inaccuracy of the rule probability, so that the correct analysis results cannot be obtained. The experimental results show that this initialization method improves the error correction performance of the model by 1.48. On the basis of the deep GLR model, a multi-layer target-attention calculation method is proposed. The GLR test method is used in the experiment to study the test of the index parameter in the single index, the corresponding GLR test statistic is established, and the statistic is proved. Asymptotically obeying the envy distribution, it not only reveals the new phenomenon in the semiparametric regression model with index terms, but also expands the application of the GLR test. Our simulation studies show that the proposed test statistic exhibits superior power. In the deep GLR task, the input and output sentences are in the same language, and the deep GLR model is a model for translating the translation problem between two different languages. By improving the deep GLR model, the simulation uses a multi-layer target-attention calculation method in the decoding end, so that the model can pay attention to the previous information multiple times and multiple weights when predicting the next word. The experimental results show that the deep GLR model with the addition of this calculation method improves the error correction performance by 1.7.

## Figures and Tables

**Table 1:** Decoding analysis of English long and short sentence vectors.

Decoding analysis name	Structural model factor	Sentence vector type	Vector set space
Verb 1	16.77	Data char type	2.3
Verb 2	16.89	Data char type	1.77
Verb 3	17.42	Data char type	1.24
User 1	18.11	Data string type	0.71
User 2	18.79	Data string type	3.28
User 3	19.31	Data string type	2.47
Pet 1	19.62	Data dictionary type	1.66
Pet 2	19.65	Data dictionary type	0.85

**Table 2:** Information flow of deep GLR model.

Information flow index	Model signal	Translation signal	Synchronization signal
Catch list = 10	43.70	28.23	24.78
Catch list = 20	11.77	10.82	9.64
Catch list = 30	32.08	22.79	13.95
Catch list = 40	48.34	27.41	5.65
Catch list = 50	39.40	18.31	23.11
Catch list = 60	46.61	32.28	30.41

**Table 3:** Semantic recognition algorithm system architecture.

Semantic recognition algorithm text	Content of simulation results
Public void actionperformed(actionevent e) {	Level for the *w*(*i*)
String dor1 = dor.gettext();	Indicates that *w*(*i*)+*w*(*j*)
If (has.containskey(number1)) {	A relatively robust growth
Has.remove(number1);	*bp*. log(*n*) in the text
Joptionpane.showmessagedialog(null, “”);	Different error distributions
Joptionpane.showmessagedialog(null, “”);	All the power cov{*w*(*i*)}
But3.addactionlistener(new {	Calculation results
String name1 = name.gettext();	Gives a better *T*(*i*, *ns*) − 1
Menu.class.getname())	*p*(*x*(*i*), *y*(*i*)) are shown

## Data Availability

The data used to support the findings of this study are available from the corresponding author upon request.
